# Supplementary material for: High-frequency, low-energy organic event-based sensors for closed-loop neurostimulation
Source: Nat Sens. 2026 Jan 15;1(1):63–72. doi: 10.1038/s44460-025-00007-x (PMC12863661; doi:10.1038/s44460-025-00007-x)
Supplement: Supplementary file 1 — Supplementary Figs. 1–20, Tables 1–3 and references. [file 44460_2025_7_MOESM1_ESM.pdf]

nature sensors

Supplementary information https://doi.org/10.1038/s44460-025-00007-x

High-frequency, low-energy organic event-based sensors for closed-loop neurostimulation

In the format provided by the authors and unedited

Supplementary Information

High-frequency, low-energy organic event-based sensors for closed-loop neurostimulation

Chi-Yuan Yang[1†], Zifang Zhao[2,3†], Han-Yan Wu[1†], Dace Gao[1], Jun-Da Huang[1], Junpeng Ji[1], Miao Xiong[1], Tiefeng Liu[1,4], Padinhare C. Harikesh[1], Adam Marks[5], Xin-Yi Wang[6], Matteo Massetti[1], Shan Shao[7], Jian Pei[6], Iain McCulloch[5,8], Magnus Berggren[1,4], Deyu Tu[1], Jennifer Gelinas[7,9], Dion Khodagholy[10]*, Simone Fabiano[1,4]*

[1]Laboratory of Organic Electronics, Department of Science and Technology, Linköping University, Norrköping, Sweden
[2]Department of Electrical Engineering, Columbia University, New York, NY, USA
[3]Department of Neurobiology and Behavior, Cornell University, Ithaca, New York, NY, USA
[4]Wallenberg Initiative Materials Science for Sustainability, Department of Science and Technology, Linköping University, Norrköping, Sweden
[5]Department of Chemistry, Chemistry Research Laboratory, University of Oxford, Oxford, UK
[6]Beijing National Laboratory for Molecular Sciences, Key Laboratory of Polymer Chemistry and Physics of the Ministry of Education, Center of Soft Matter Science and Engineering, College of Chemistry and Molecular Engineering, Peking University, Beijing, China
[7]Department of Pediatrics, University of California, Irvine, Irvine, CA, USA
[8]Andlinger Center for Energy and the Environment and Department of Electrical and Computer Engineering, Princeton University, Princeton, NJ, USA
[9]Department of Neurobiology and Anatomy, University of California, Irvine, Irvine, CA, USA
[10]Department of Electrical Engineering, University of California, Irvine, Irvine, CA, USA

[†]These authors contributed equally to this work.
Email: dion.kh@uci.edu, simone.fabiano@liu.se

Table of Contents:

Supplementary Figures 1-20

Supplementary Tables 1-3

Supplementary References

Supplementary Figures 1-20

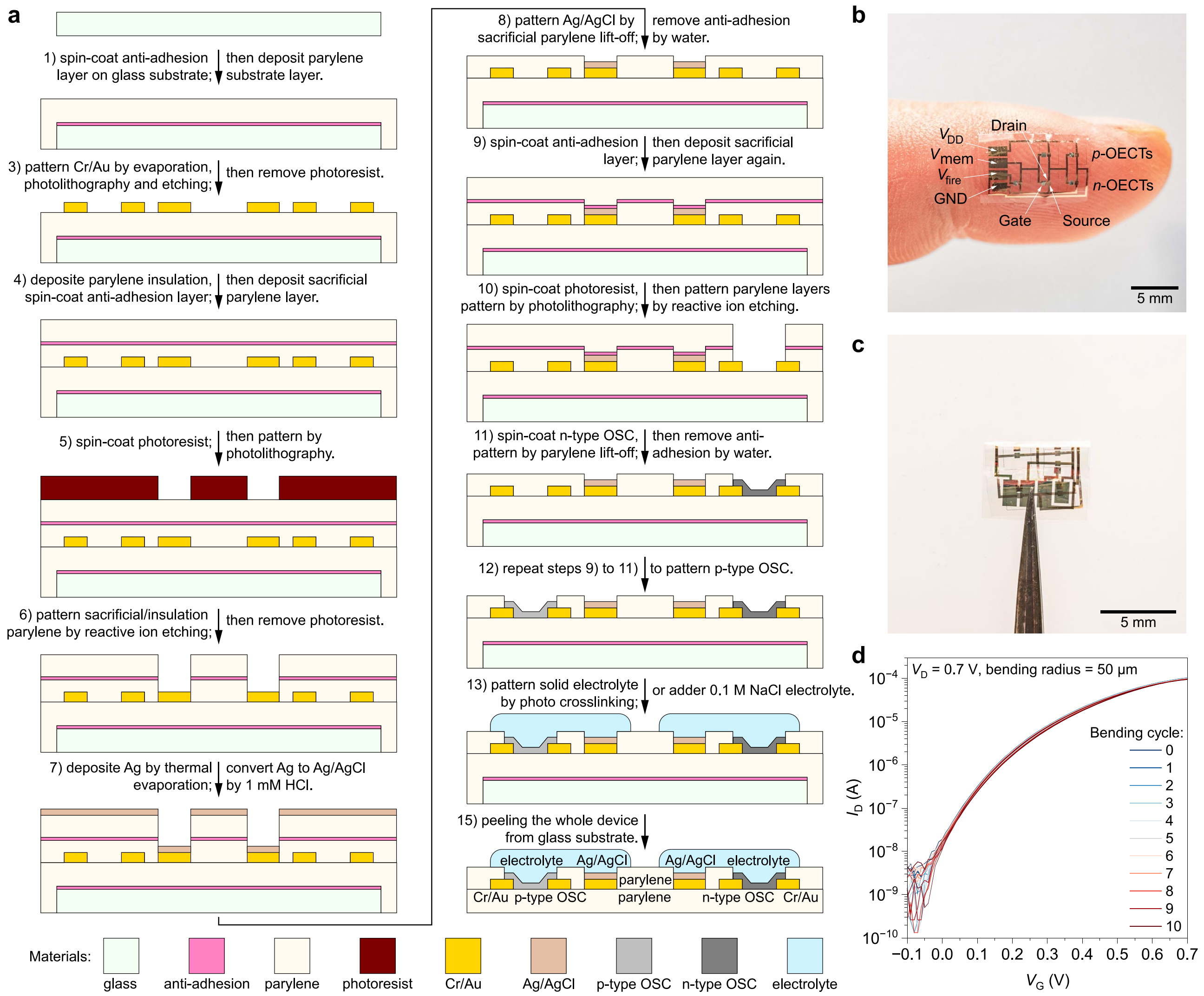

Supplementary Fig. 1 | Device fabrication. a, Illustration of the OECN fabrication process on Parylene C substrate. **b-c**, Photograph and detailed illustration of the conformable OECN circuit on a human finger (**b**) and on the tip of tweezers (**c**). **d**, Transfer curve of conformable BBL-based OECT after bending for 10 cycles (bending radius = 50 µm).

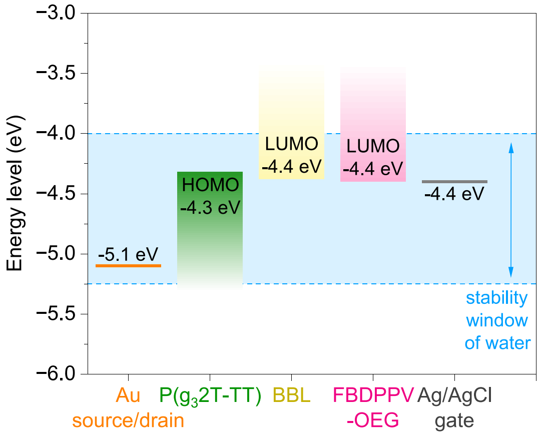

Supplementary Fig. 2 | Energy levels. a, HOMO/LUMO energy levels of OECT channel materials used in this study, along with the Fermi levels of the source/drain and gate electrodes, and the stability window of water. The HOMO level of P($g_3$2T-TT) and the LUMO levels of BBL and FBDPPV-OEG are well aligned with the electrode Fermi levels, consistent with the small V_{th} observed in the OECTs.

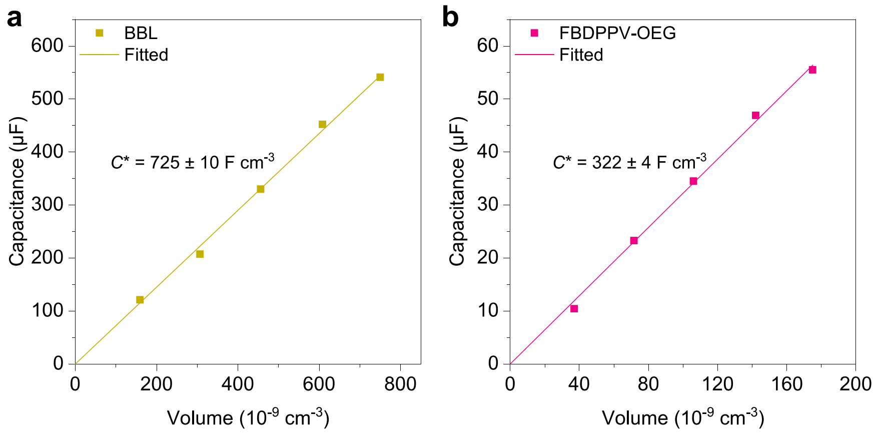

Supplementary Fig. 3 | Volumetric capacitance of the n-type polymers. a-b, Volume-dependent capacitance of (**a**) BBL and (**b**) FBDPPV-OEG. The capacitance was measured using electrochemical impedance spectroscopy (EIS) at a voltage of –0.7 V. The volumetric capacitance was obtained from the linear fit of the volume-dependent data. FBDPPV-OEG exhibits more than two times lower C^* compared to BBL.

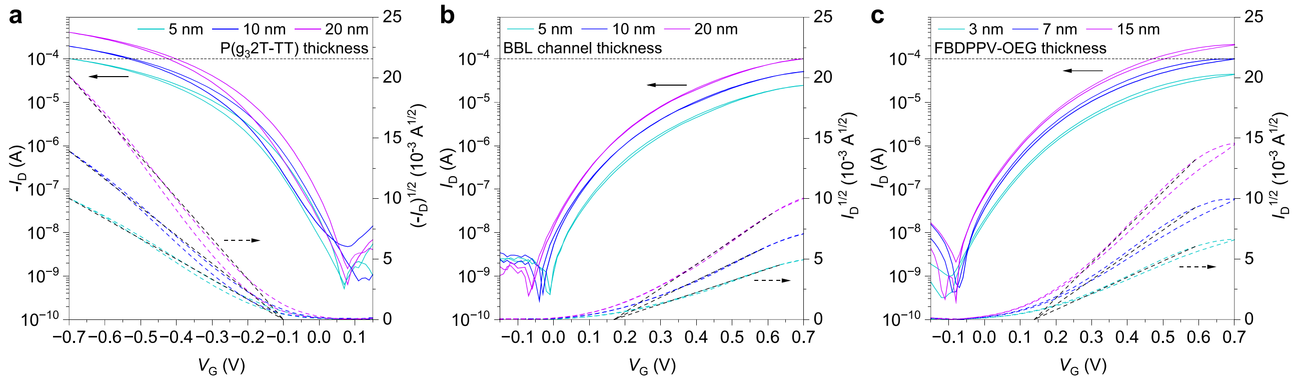

Supplementary Fig. 4 | Effect of channel thickness. a, Channel thickness-dependent transfer curves OECTs based on (**a**) P($g_3$2T-TT), (**b**) BBL, and (**c**) FBDPPV. At optimized channel thicknesses (5 nm for P($g_3$2T-TT), 20 nm for BBL, and 7 nm for FBDPPV-OEG), the p-type and n-type OECTs exhibited well-balanced charge transport.

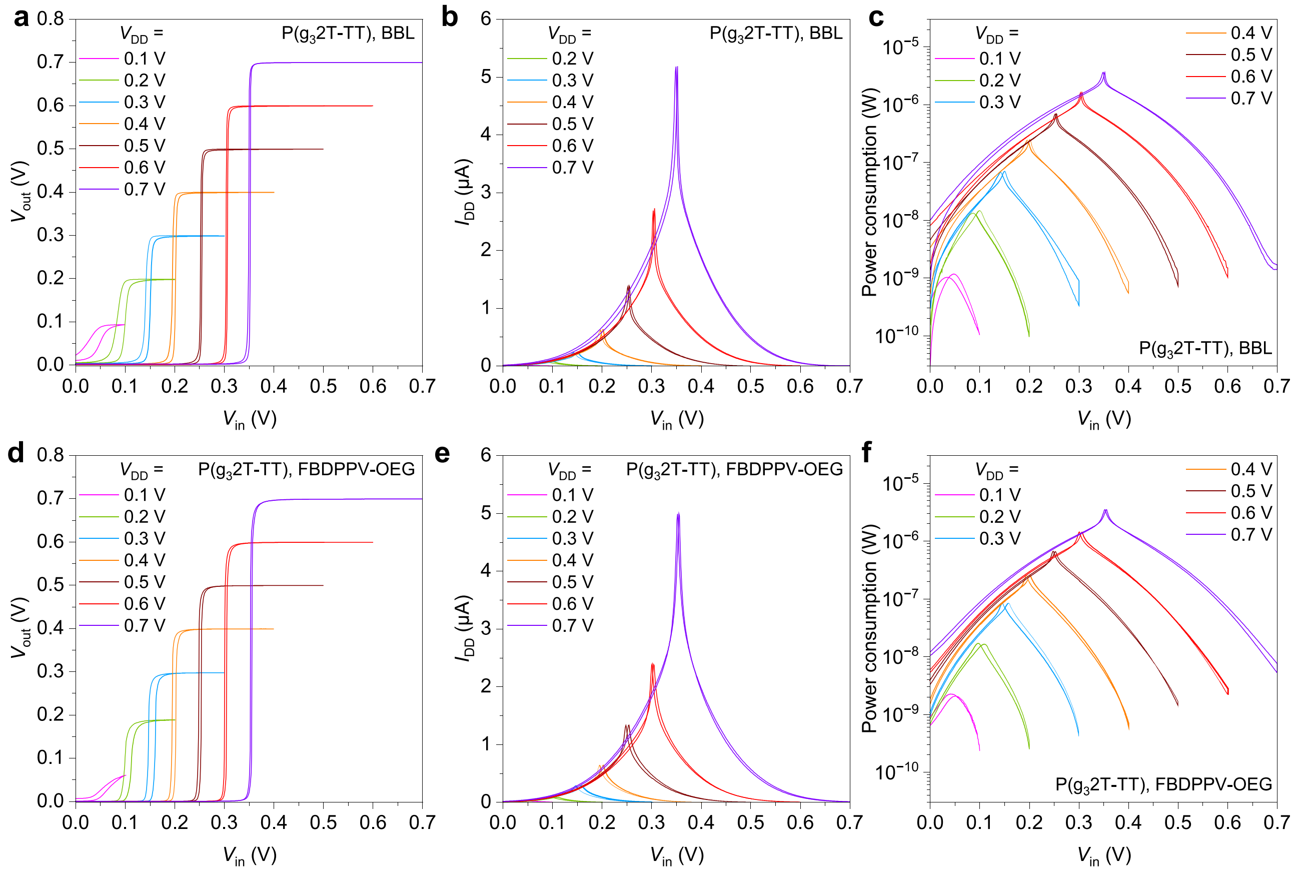

Supplementary Fig. 5 | Non-inverting amplifier performance. a-c, Voltage transfer characteristics (**a**), I_{DD} (**b**), and power consumption (**c**) of the non-inverting amplifier (L = 3.6 μm) based on P($g_3$2T-TT)/BBL. **d-f**, Voltage transfer characteristics (**d**), I_{DD} (**e**), and power consumption (**f**) of the non-inverting amplifier (L = 3.6 μm) based on P($g_3$2T-TT)/FBDPPV-OEG.

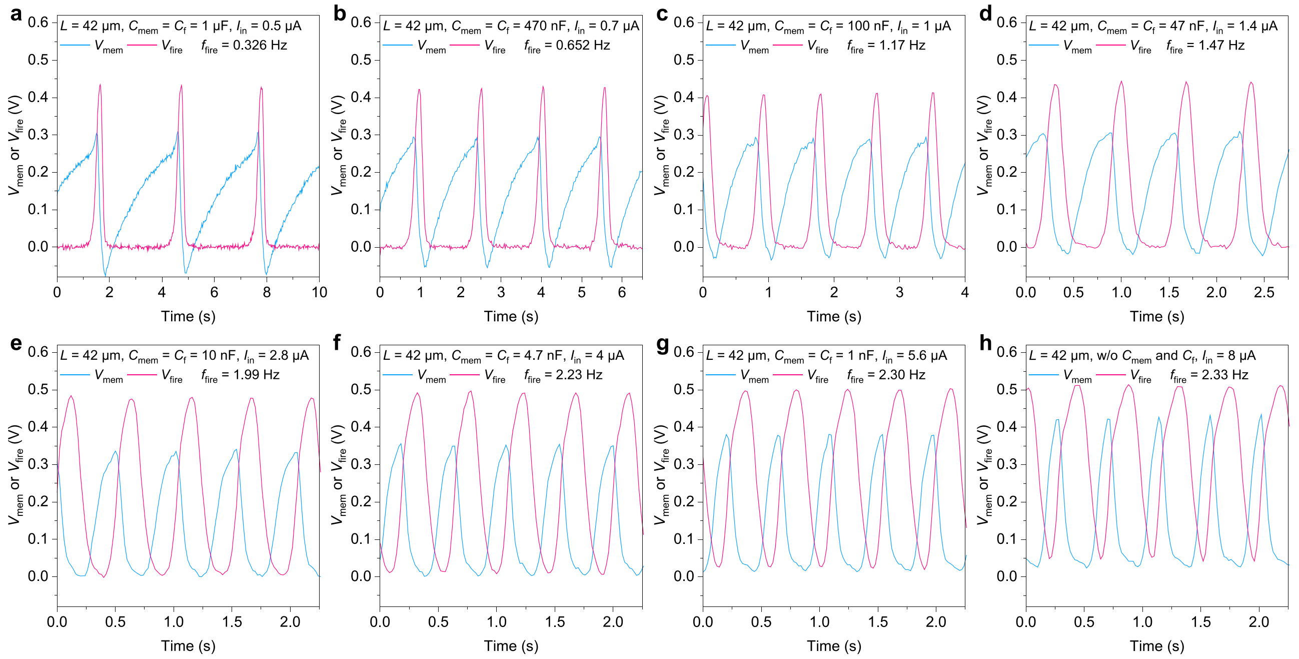

Supplementary Fig. 6 | OECN spiking performance. a-h, Spiking behavior of OECNs based on P(g$_3$2T-TT)/BBL (L = 42 μm) under various input currents (I_{in}) and capacitances (C_{mem} and C_f), with firing frequencies ranging from 0.326 to 2.33 Hz (V_{DD} = 0.6 V).

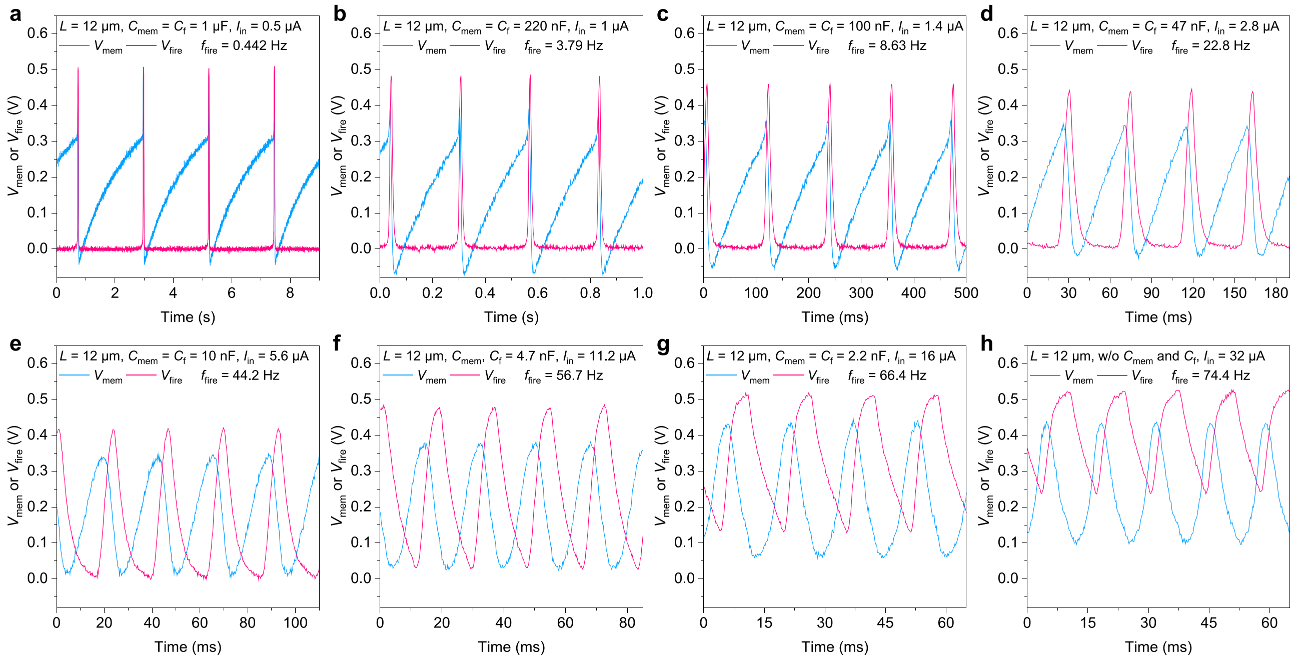

Supplementary Fig. 7 | OECN spiking performance. a-h, Spiking behavior of OECNs based on P(g$_3$2T-TT)/BBL (L = 12 μm) under various input currents (I_{in}) and capacitances (C_{mem} and C_f), with firing frequencies ranging from 0.442 to 74.4 Hz (V_{DD} = 0.6 V).

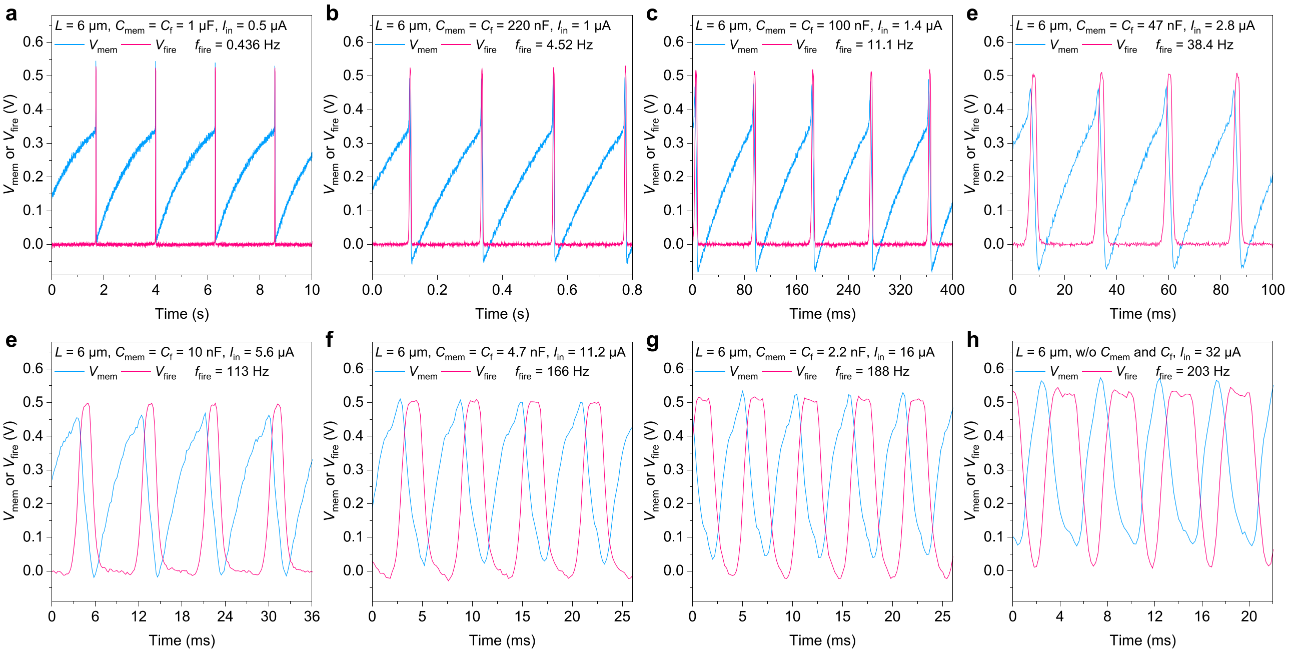

Supplementary Fig. 8 | OECN spiking performance. a-h, Spiking behavior of OECNs based on P(g$_3$2T-TT)/BBL (L = 6 μm) under various input currents (I_{in}) and capacitances (C_{mem} and C_f), with firing frequencies ranging from 0.438 to 203 Hz (V_{DD} = 0.6 V).

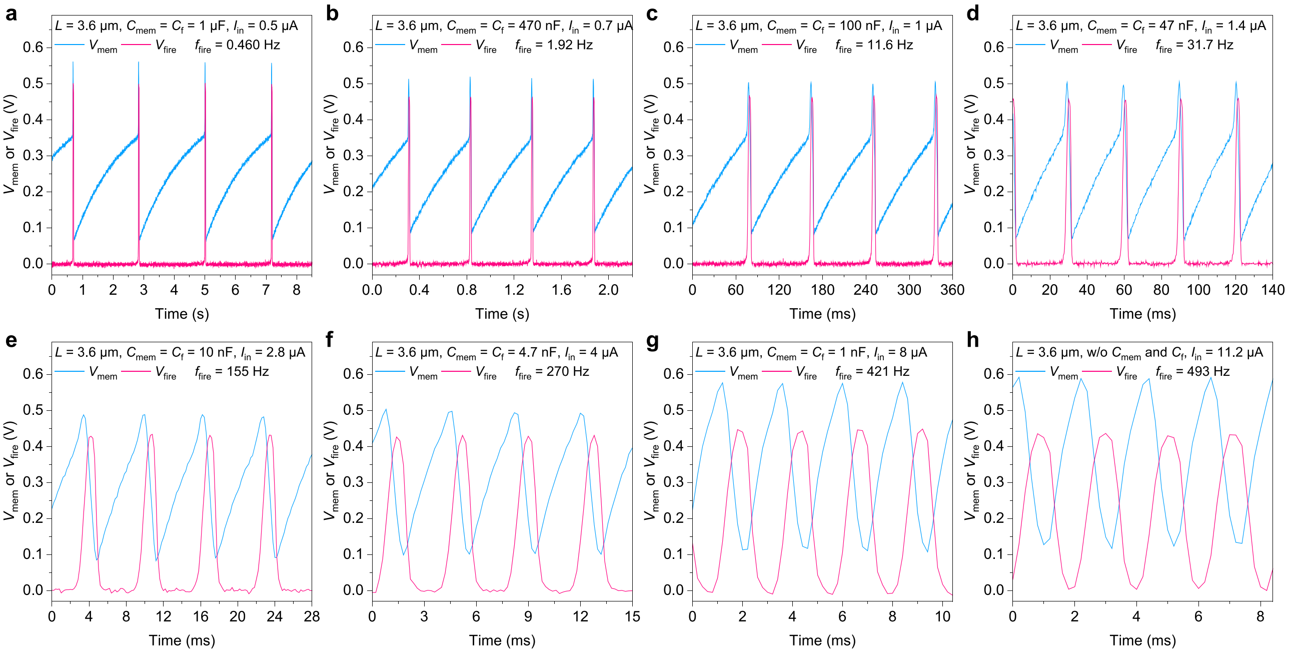

Supplementary Fig. 9 | OECN spiking performance. a-h, Spiking behavior of OECNs based on P(g$_3$2T-TT)/BBL (L = 3.6 μm) under various input currents (I_{in}) and capacitances (C_{mem} and C_f), with firing frequencies ranging from 0.460 to 493 Hz (V_{DD} = 0.6 V).

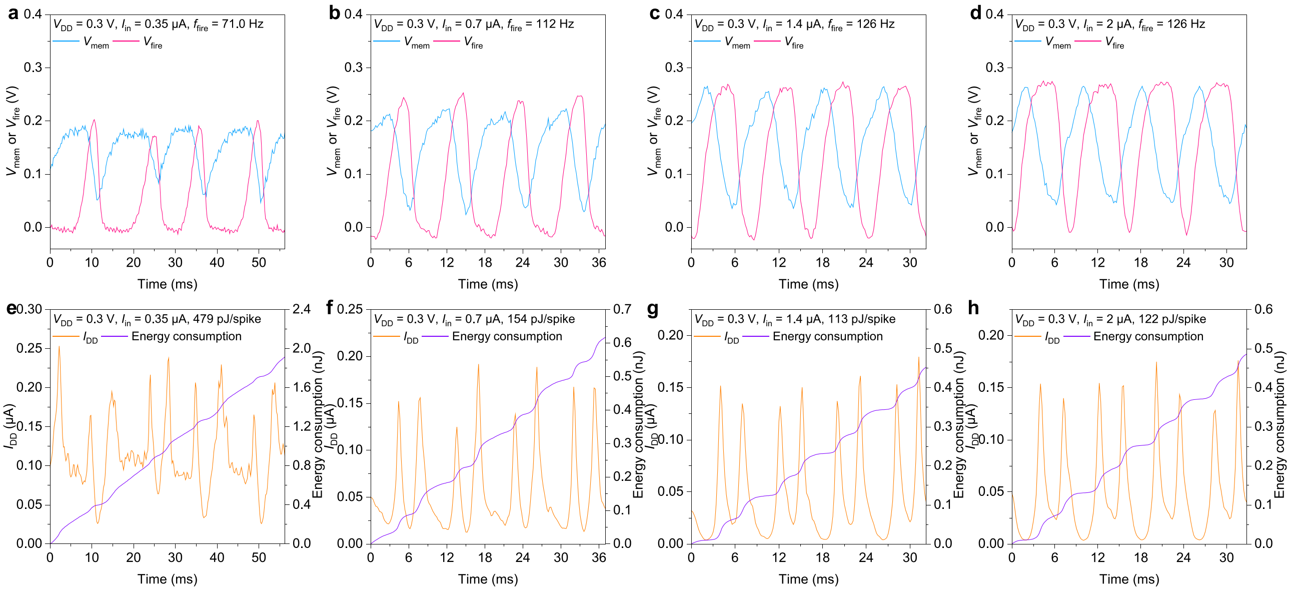

Supplementary Fig. 10 | OECN energy consumption. a-d, Spiking behavior of OECNs based on P(g$_3$2T-TT)/BBL (L = 3.6 μm, without C_{mem} and C_f, V_{DD} = 0.3 V) under various input currents and corresponding energy consumption (**e-f**).

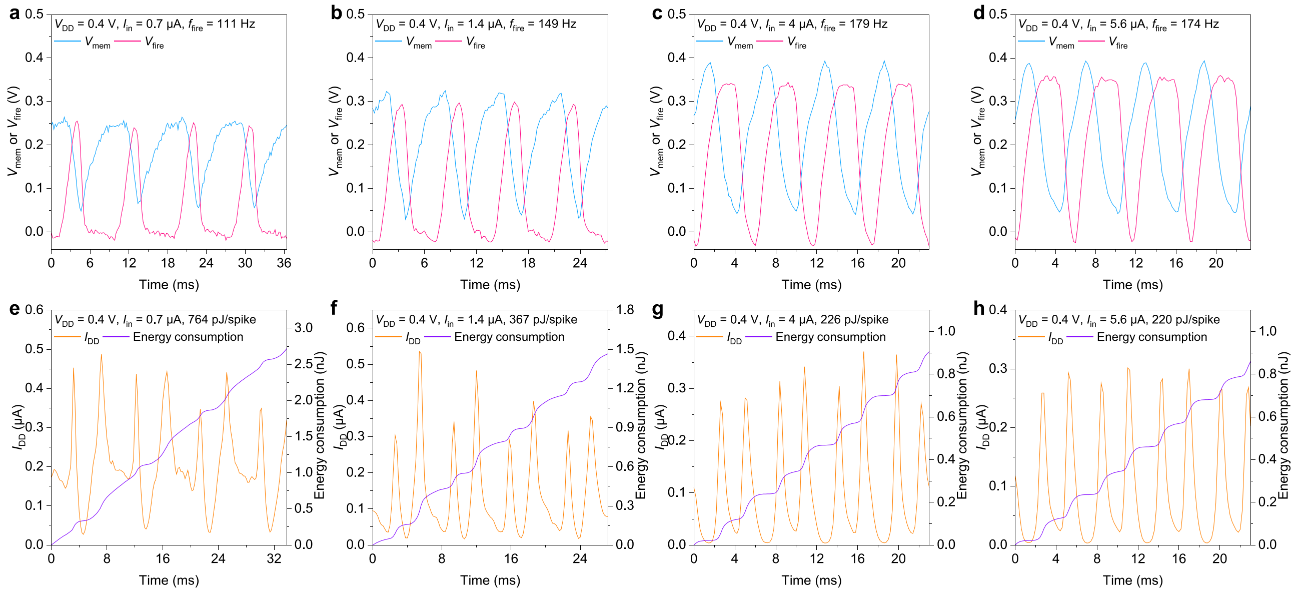

Supplementary Fig. 11 | OECN energy consumption. a-d, Spiking behavior of OECNs based on P(g$_3$2T-TT)/BBL (L = 3.6 μm, without C_{mem} and C_f, V_{DD} = 0.4 V) under various input currents and corresponding energy consumption (**e-f**).

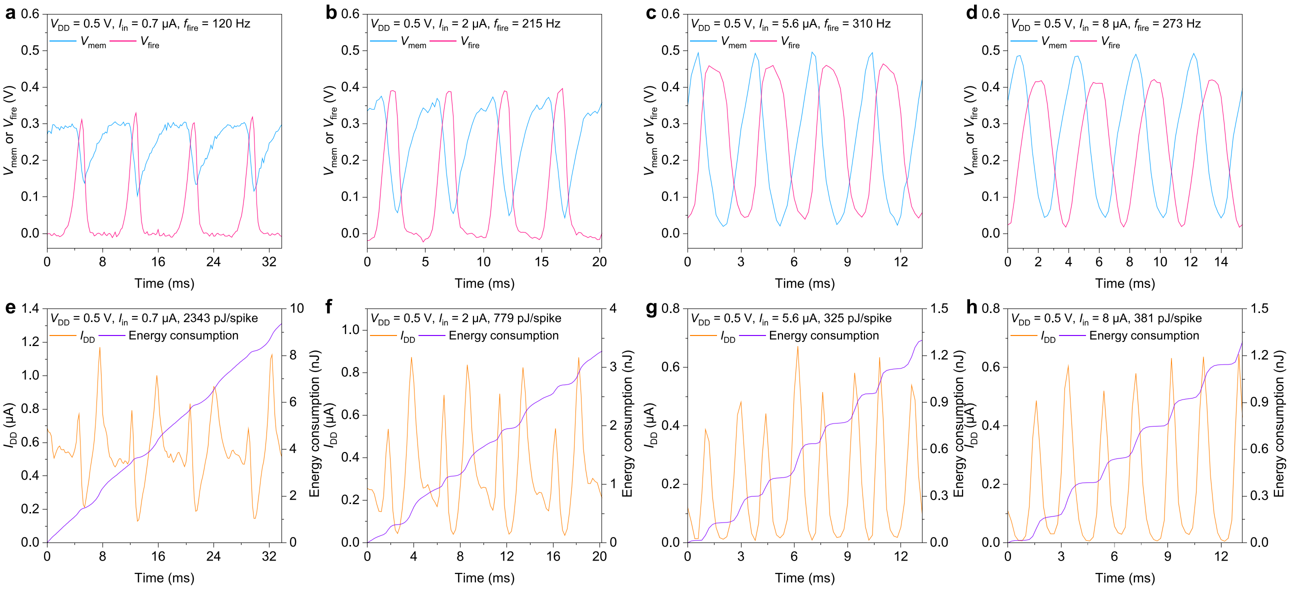

Supplementary Fig. 12 | OECN energy consumption. a-d, Spiking behavior of OECNs based on P(g$_3$2T-TT)/BBL (L = 3.6 μm, without C_{mem} and C_{f}, V_{DD} = 0.5 V) under various input currents and corresponding energy consumption (**e-f**).

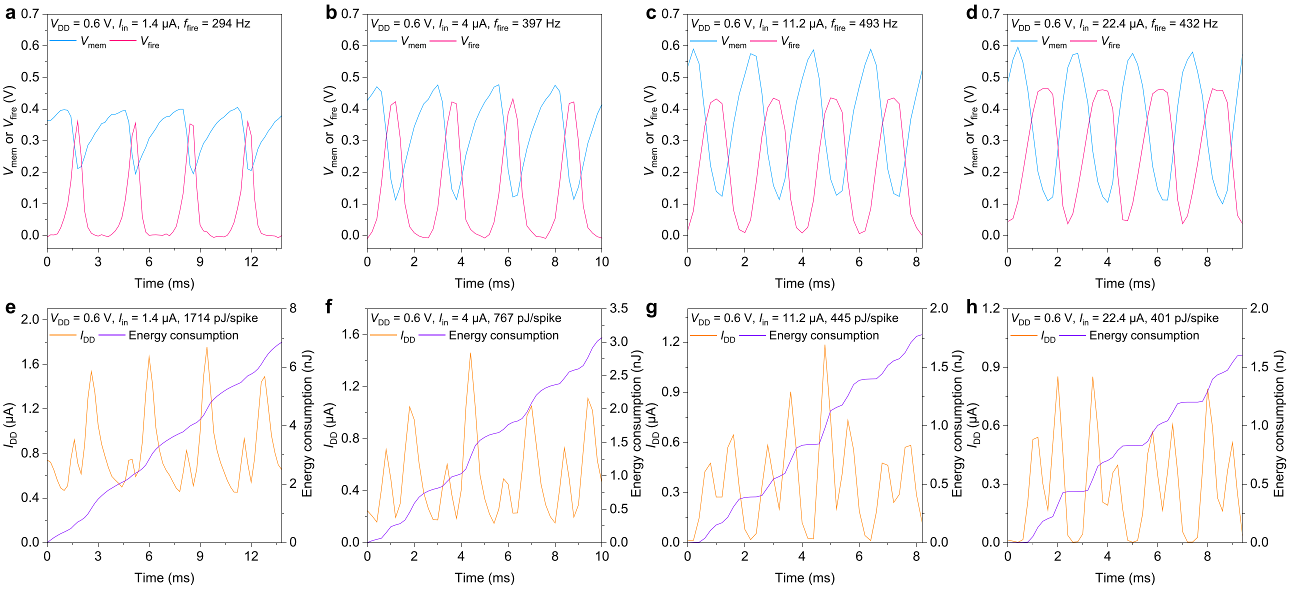

Supplementary Fig. 13 | OECN energy consumption. a-d, Spiking behavior of OECNs based on P(g$_3$2T-TT)/BBL (L = 3.6 μm, without C_{mem} and C_{f}, V_{DD} = 0.6 V) under various input currents and corresponding energy consumption (**e-f**).

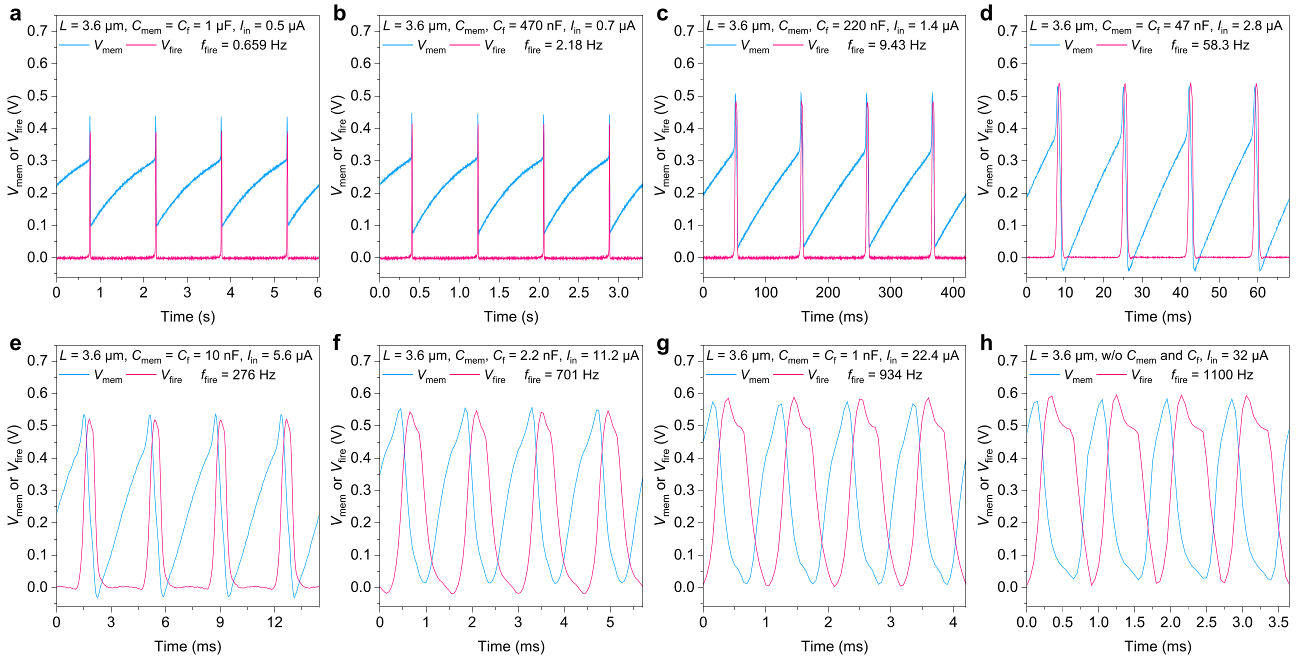

Supplementary Fig. 14 | OECN spiking performance. a-h, Spiking behavior of OECNs based on P(g$_3$2T-TT)/FBDPPV-OEG (L = 3.6 μm) under various input currents (I_{in}) and capacitances (C_{mem} and C_f), with firing frequencies ranging from 0.659 to 1100 Hz (V_{DD} = 0.6 V).

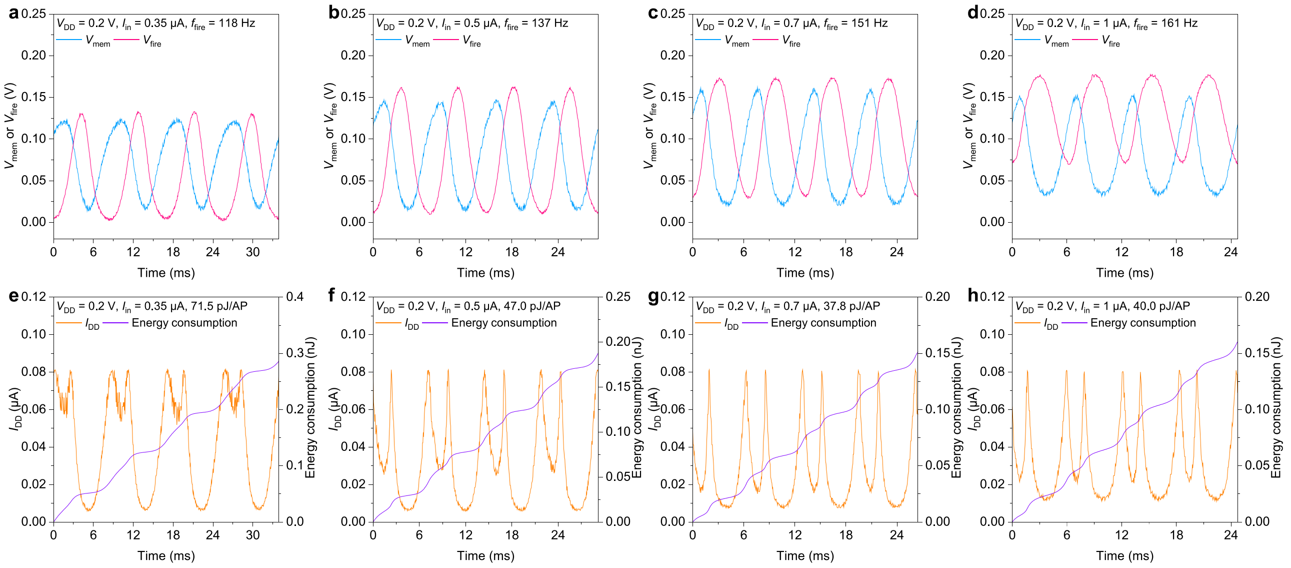

Supplementary Fig. 15 | OECN energy consumption. a-d, Spiking behavior of OECNs based on P(g$_3$2T-TT)/FBDPPV-OEG (L = 3.6 μm, without C_{mem} and C_f, V_{DD} = 0.2 V) under various input currents and corresponding energy consumption (**e-f**).

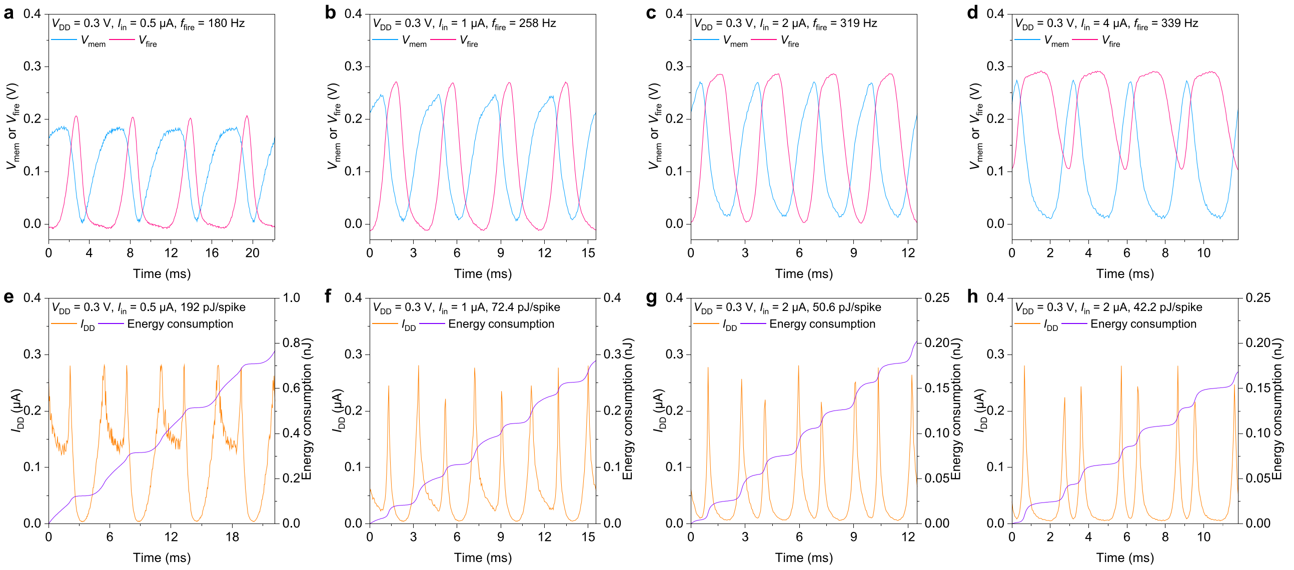

Supplementary Fig. 16 | OECN energy consumption. a-d, Spiking behavior of OECNs based on P(g$_3$2T-TT)/FBDPPV-OEG (L = 3.6 μm, without C_{mem} and C_{f}, V_{DD} = 0.3 V) under various input currents and corresponding energy consumption (**e-f**).

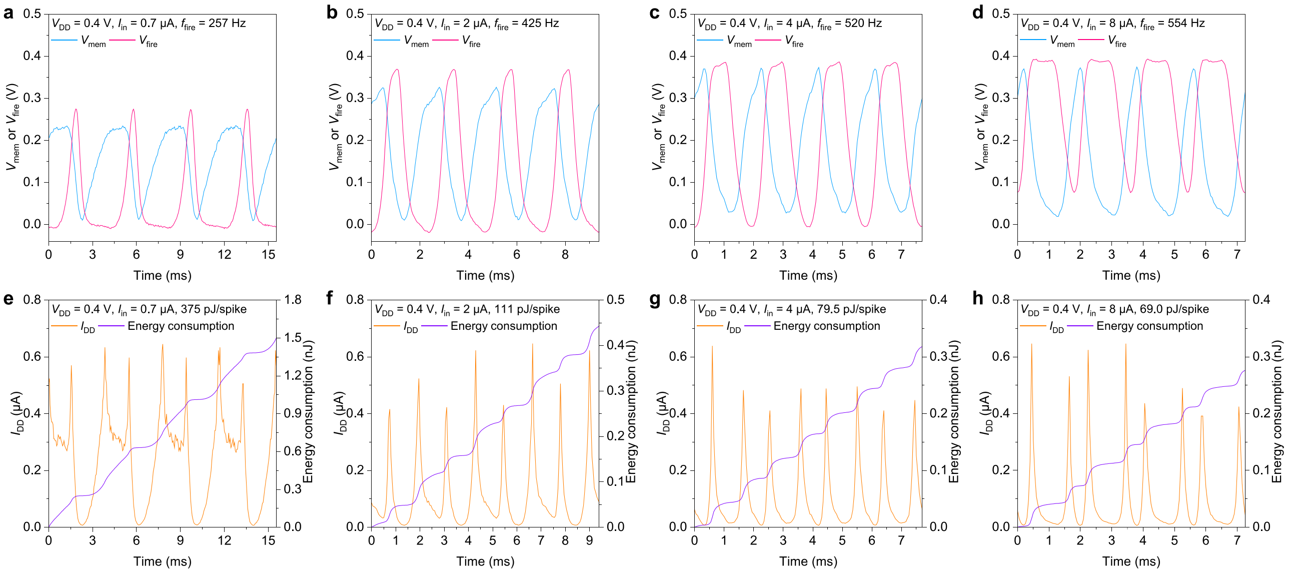

Supplementary Fig. 17 | OECN energy consumption. a-d, Spiking behavior of OECNs based on P(g$_3$2T-TT)/FBDPPV-OEG (L = 3.6 μm, without C_{mem} and C_{f}, V_{DD} = 0.4 V) under various input currents and corresponding energy consumption (**e-f**).

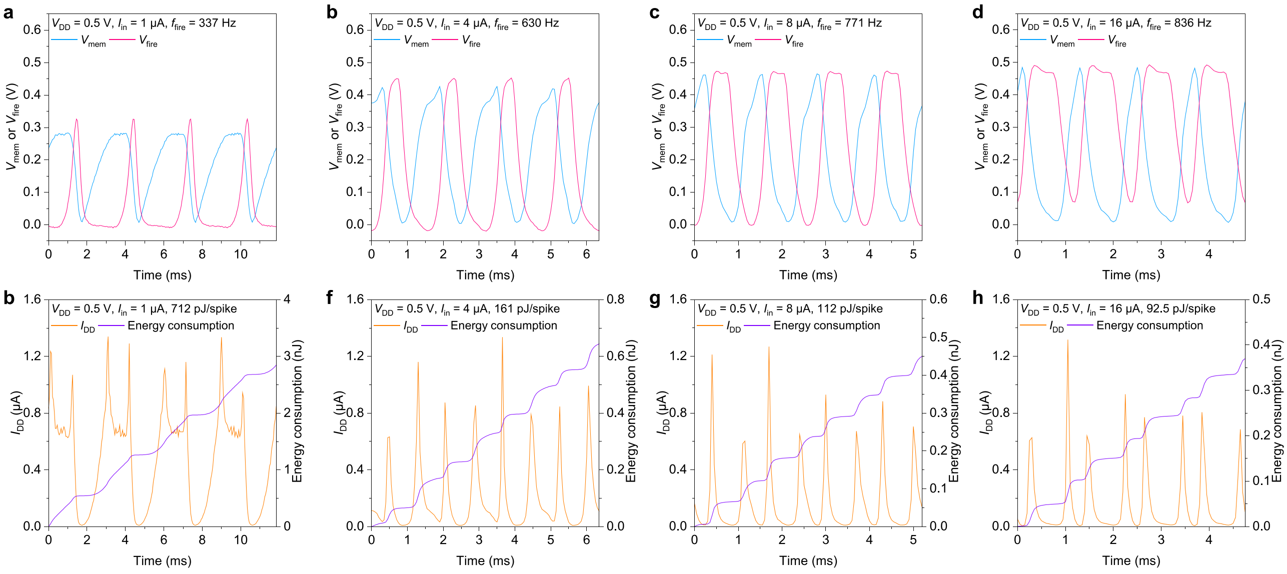

Supplementary Fig. 18 | OECN energy consumption. a-d, Spiking behavior of OECNs based on P(g₃2T-TT)/FBDPPV-OEG (L = 3.6 μm, without C_{mem} and C_f, V_{DD} = 0.5 V) under various input currents and corresponding energy consumption (**e-f**).

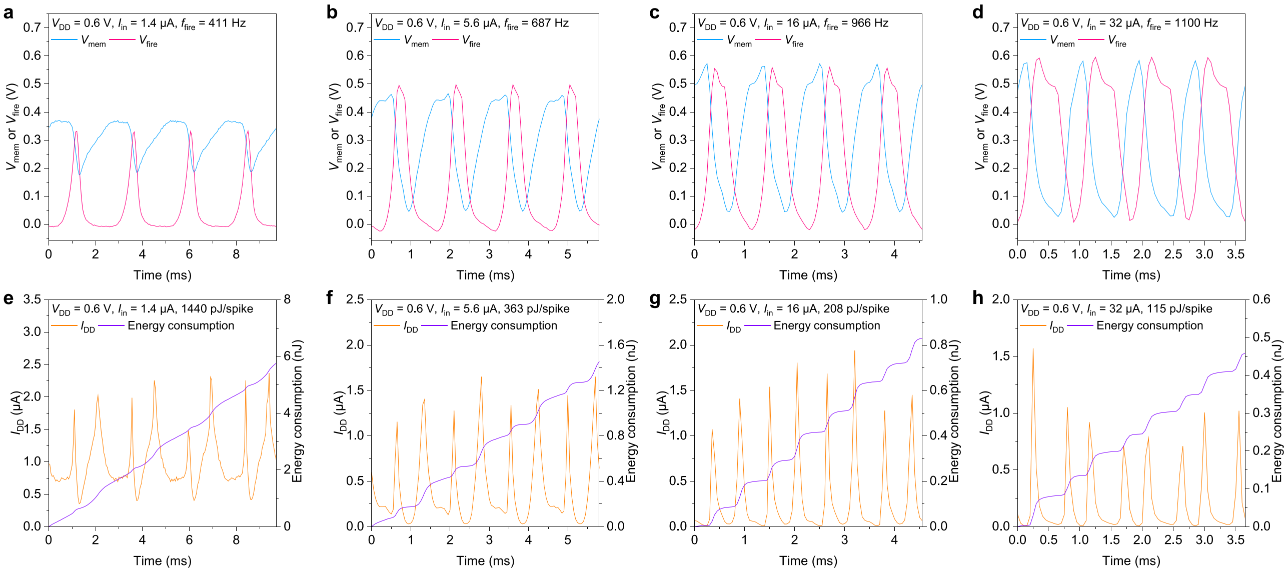

Supplementary Fig. 19 | OECN energy consumption. a-d, Spiking behavior of OECNs based on P(g₃2T-TT)/FBDPPV-OEG (L = 3.6 μm, without C_{mem} and C_f, V_{DD} = 0.6 V) under various input currents and corresponding energy consumption (**e-f**).

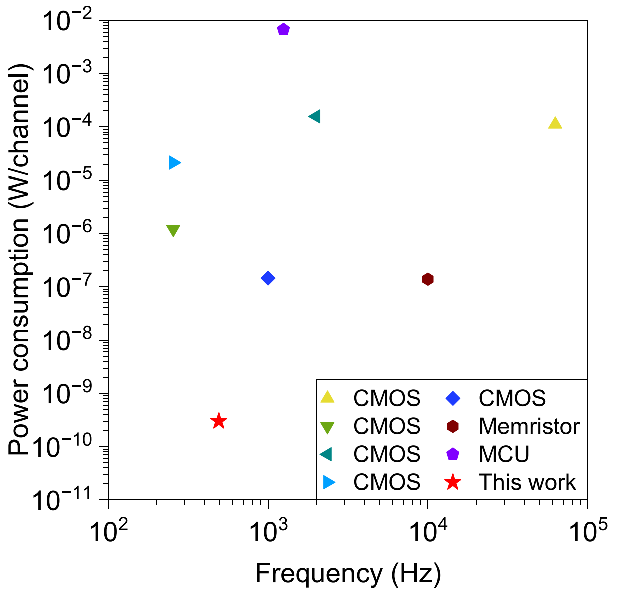

Supplementary Fig. 20 | Power consumption of neural signal processors. Comparison of OECN power consumption with other closed-loop neural signal processors, including CMOS SoCs, MCUs, and memristor-based systems. Detailed data and references are provided in Supplementary Table 3.

Supplementary Tables 1-3

Supplementary Table 1. Survey of n-type OECT materials. Summary of high-performance n-type channel materials used in planar n-type accumulation-mode OECTs.

Materials	V_{th} [V]	$g_{m,norm}$ [S cm^{-1}]	μC^* [F cm^{-1} V^{-1} s^{-1}]	τ_{ON} [ms]	Year	Refs.
p(gNDI-gT2)	0.28	0.1085	NA	5	2016	[1]
P-50	0.36	0.067	NA	NA	2018	[2]
P-75	0.29	0.141	NA	NA	2018	[2]
P-90	0.26	0.21	NA	NA	2018	[2]
P-100	0.25	0.204	NA	NA	2018	[2]
C60-TEG	0.55	0.0146	7	80	2019	[3]
P90, PFBT	0.29	0.0111	0.0008	NA	2019	[4]
P90, MBT	0.27	0.0059	0.0023	NA	2019	[4]
p(NDI-T2-L2)	0.22	0.0084	0.046	40	2020	[5]
P-90	0.25	0.0113	NA	NA	2020	[6]
P-90:TBAF(10%)	0.25	0.0299	NA	NA	2020	[6]
P-90:TBAF(40%)	0.22	0.0905	NA	24	2020	[6]
P-90:TBAF(80%)	0.25	0.0833	NA	NA	2020	[6]
f-BTI2TEG-T	0.68	0.26	2.13	332	2021	[7]
f-BTI2TEG-FT	0.53	4.42	14.71	272	2021	[7]
BBL	0.19	0.815	1.99	5.2	2021	[8]
P90	0.24	0.009	0.0343	41	2021	[8]
PgNaN	0.37	0.212	0.662	127	2021	[9]
PgNgN	0.21	0.007	0.037	NA	2021	[9]
p(gNDI-gT2)	0.26	0.13	0.06	NA	2021	[10]
p(C3-gNDI-gT2)	0.25	0.34	0.13	NA	2021	[10]
p(C6-gNDI-gT2)	0.37	0.37	0.16	NA	2021	[10]
p(C4-T2-OMe)	0.46	0.10	0.07	NA	2021	[11]
p(C4-T2-C0-EG)	0.32	0.31	0.22	24.6	2021	[11]
p(C_4-T2-C_2-EG)	0.30	0.02	0.01	6.2	2021	[11]
p(C_4-T2-C_4-EG)	0.33	0.01	0.006	12.5	2021	[11]
p(C_2-T2)	0.27	0.40	0.2	6.3	2021	[11]
p(C_4-T2)	0.24	0.63	0.3	7.5	2021	[11]
p(C_6-T2)	0.30	2.28	1.29	9.6	2021	[11]
p(C_8-T2)	0.37	0.15	0.13	12.7	2021	[11]
2DPP-OD-TEG	0.89	0.73	7	500	2021	[12]
f-BTI2g-TVT	0.9	0.27	1.50	68	2022	[13]
f-BTI2g-TVTCN	0.68	12.8	41.3	52	2022	[13]
P(gTDPP2FT)	0.64	0.52	54.8	1.75	2022	[14]
AIG-BT	0.55	0.029	0.12	NA	2022	[15]
p(C-T)	0.43	0.80	6.7	NA	2022	[16]
p(N-T)	0.25	0.72	4.3	NA	2022	[16]
p(C-2T)	0.44	0.14	1.0	NA	2022	[16]
PBBTL:BBL	0.26	0.43	1.36	1.72	2022	[17]
**BBL**	**0.15**	**11.1**	**25.9**	**0.38**	**2022**	**[18]**
gNDI-EDBT	0.52	0.3	0.02	377.6	2022	[19]
gNDI-BT	0.30	1.9	0.09	102.3	2022	[19]
gNDI-FBT	0.19	3.6	0.12	45.5	2022	[19]
p(C-V)	0.17	1.81	14.89	1.201	2023	[20]

p(C2F-V)	0.02	25.67	107.56	0.336	2023	[20]
P75	0.28	0.03	0.01	161.6	2023	[21]
P75-TBAClO4	0.29	0.39	0.24	7.4	2023	[21]
P75-TBAPF6	0.29	0.21	0.11	7.5	2023	[21]
P75-LiClO4	0.30	0.22	0.09	6.0	2023	[21]
gNDI-V	0.3	0.42	2.31	NA	2023	[22]
gNDI-T	0.2	0.11	0.42	NA	2023	[22]
CNg4T2-CNT2	0.75	6.75	27.01	102	2023	[23]
lgTNR	0.35	0.97	4.2	308	2023	[24]
bgTNR	0.29	7.10	31.6	489	2023	[24]
t-gdiPDI	-0.07	0.055	0.21	123	2023	[25]
d-gdiPDI	0.11	0.101	0.33	87	2023	[25]
3gDNR	0.27	2.16	9.4	439	2023	[26]
4gDNR	0.25	1.17	4.7	207	2023	[26]
TDPP-CN-G7	0.16	NA	0.68	NA	2023	[27]
TDPP-RD-G7	0.34	NA	5.43	10.5	2023	[27]
f-BTI2g-TVT	0.97	1.60	6.96	NA	2024	[28]
f-BTI2g-TVTF	0.75	22.6	90.2	NA	2024	[28]
f-BTI2g-TVTCl	0.79	3.97	18.9	NA	2024	[28]
f-BTI2g-SVSCN	0.70	48.3	150.9	7.3	2024	[29]
f-BseI2g-SVSCN	0.68	71.4	191.2	5.1	2024	[29]
p(N-T)	0.25	0.72	4.3	NA	2024	[30]
p(N-T):PS 10K 1:6	0.27	1.6	13.4	NA	2024	[30]
p(N-T):PMM 10K 1:6	0.25	0.7	5.3	NA	2024	[30]
p(N-T):PS 1K 1:6	0.25	0.6	4.9	NA	2024	[30]
Cl_2-BAL	0.26	1.63	6.20	2.4	2024	[31]
Cl_4-BAL	0.32	0.21	0.83	3.1	2024	[31]
o-CNgTVT-2FT	0.79	17.4	88.2	2.86	2025	[32]
**FBDPPV-OEG**	**0.4**	**22**	**40**	**0.1**	**2025**	**This work**

Supplementary Table 2. Survey of p-type OECT materials. Summary of high-performance p-type channel materials used in planar p-type accumulation-mode OECTs.

**Materials**	V_{th} **[V]**	$g_{m,norm}$ **[S cm^{-1}]**	μC^* **[F cm^{-1} V^{-1} s^{-1}]**	τ_{ON} **[ms]**	**Year**	**Refs.**
PTHS	-0.4	5.67	NA	0.4	2014	[33]
**P($g_3$2T-TT)**	**-0.1**	**135**	**227**	**0.42**	**2016**	**[34]**
g2T-T	-0.2	NA	167	1.4	2017	[35–37]
Crys-P	NA	4100	490	NA	2018	[38]
ProDOT(OE)-DMP	-0.45	NA	6.99	NA	2018	[39]
P3MEEMT	-0.42	NA	96.7	NA	2019	[40]
PIBET-AO	-0.44	1.3	NA	654	2019	[41]
PIBET-O	-0.44	1.0	NA	714	2019	[41]
PIBET-BO	-0.62	0.8	NA	862	2019	[41]
PIBET-A	-0.86	0.5	NA	29000	2019	[41]
PIBT-BO	-0.80	1.4	NA	3500	2019	[41]
PTHS−TMA+-co-P3HT	-0.15	70.49	1.7	0.4	2019	[42]
P(g2T2-g4T2)	0.02	NA	522	NA	2020	[43]
p(gPyDPP-MeOT2)	-0.35	19.5	1.8	0.77	2020	[44]
g-0%	-0.39	0.00125	0.001	3.2	2020	[45]
g-50%	-0.27	0.87	0.87	2.9	2020	[45]

g-75%	-0.21	129.09	78	2.3	2020	[45]
g-100%	-0.08	341.82	163	0.13	2020	[45]
2g	-0.08	23.64	16	0.14	2020	[45]
P(lgDPP-MeOT2)	-0.17	7.04	174	0.578	2021	[46]
P(bgDPP-MeOT2)	-0.33	5.33	195	0.516	2021	[46]
TDPP-gTVT	-0.36	47.8	205.2	7.3	2021	[47]
TDPP-gTBTT	-0.42	3.65	21.5	8.7	2021	[47]
PProDOT-DPP $LiPF_6$	+0.10	NA	310	260	2021	[48]
Pg$_3$BTTT	-0.24	194	502	NA	2021	[49]
P(gDPP-T2)	-0.52	NA	342	NA	2021	[50]
PTDPP-DT	-0.93	19	149	NA	2021	[51]
PDPP[T]$_2$(TEG)-EDOT	–0.38	0.53	14	11	2021	[52]
PDPP[T]2(TEG)3-MEET	–0.36	0.51	45	6	2021	[52]
PBBT-H	-0.36	1.11	2.52	0.738	2022	[53]
PBBT-Me	-0.52	40.6	92.3	3.19	2022	[53]
P(gTDPPT)	-0.62	1.18	45.9	0.46	2022	[14]
PBBTL	-0.41	0.58	4.82	2.89	2022	[17]
P3APPT	-0.27	NA	41.3	9000	2022	[54]
P(g$_3$2T-TT)	-0.06	130	556	NA	2022	[55]
P3gCPDT-2gT2	–0.24	38.3	92.0	22.8	2022	[56]
P3gCPDT-1gT2	–0.21	168.3	288.6	36.7	2022	[56]
P3gCPDT-MeOT2	–0.09	344.0	448.6	49.3	2022	[56]
g2T2-gBT2	-0.17	16.3	40	93	2023	[57]
g2T2-gBT4	-0.10	154.9	359	50	2023	[57]
g2T2-gBT6	-0.10	92.4	203	37	2023	[57]
g4T2-T2	-0.21	38.75	133.62	971	2023	[23]
g4T2-CNT2	-0.74	0.77	4.84	471	2023	[23]
CNg4T2-T2	-0.83	0.89	12.73	981	2023	[23]
Aligned PBTTT-^{8}O	-0.32	2580	10660	820	2025	[58]
Pristine PBTTT-8O	-0.32	430	960	1670	2025	[58]
gFBT-g2T	-0.33	298	826	19	2025	[59]

Supplementary Table 3. Power consumption data of OECNs and other neural signal processors. Comparison of OECN power consumption with other closed-loop neural signal processors, including CMOS SoCs, MCUs, and memristor-based systems.

Neural signal processor	Frequency [Hz]	Channel	Power consumption [W/channel]	Refs.
CMOS SoC	62500	8	1.1×10^{-4}	[60]
CMOS SoC	256	8	1.2×10^{-6}	[61]
CMOS SoC	2000	16	1.6×10^{-4}	[62]
CMOS SoC	256	32	2.1×10^{-5}	[63]
CMOS SoC	1000	8	1.5×10^{-7}	[64]
Memristor	10000	1	1.4×10^{-7}	[65]
MCU	1250	1	6.6×10^{-3}	[66]
OECN	493	1	3.0×10^{-10}	This work

Supplementary References

1. Giovannitti, A. *et al.* N-type organic electrochemical transistors with stability in water. *Nat. Commun.* **7**, 13066 (2016).
2. Giovannitti, A. *et al.* The Role of the Side Chain on the Performance of N-type Conjugated Polymers in Aqueous Electrolytes. *Chem. Mater.* **30**, 2945–2953 (2018).
3. Bischak, C. G., Flagg, L. Q., Yan, K., Li, C.-Z. & Ginger, D. S. Fullerene Active Layers for n-Type Organic Electrochemical Transistors. *ACS Appl. Mater. Interfaces* **11**, 28138–28144 (2019).
4. Paterson, A. F. *et al.* On the Role of Contact Resistance and Electrode Modification in Organic Electrochemical Transistors. *Adv. Mater.* **31**, 1902291 (2019).
5. Kawan, M. *et al.* Monitoring supported lipid bilayers with n-type organic electrochemical transistors. *Mater. Horiz.* **7**, 2348–2358 (2020).
6. Paterson, A. F. *et al.* Water stable molecular n-doping produces organic electrochemical transistors with high transconductance and record stability. *Nat. Commun.* **11**, 3004 (2020).
7. Feng, K. *et al.* Fused Bithiophene Imide Dimer-Based n-Type Polymers for High-Performance Organic Electrochemical Transistors. *Angew. Chem. Int. Ed.* **60**, 24198–24205 (2021).
8. Surgailis, J. *et al.* Mixed Conduction in an N-Type Organic Semiconductor in the Absence of Hydrophilic Side-Chains. *Adv. Funct. Mater.* **31**, 2010165 (2021).
9. Chen, X. *et al.* n-Type Rigid Semiconducting Polymers Bearing Oligo(Ethylene Glycol) Side Chains for High-Performance Organic Electrochemical Transistors. *Angew. Chem. Int. Ed.* **60**, 9368–9373 (2021).
10. Maria, I. P. *et al.* The Effect of Alkyl Spacers on the Mixed Ionic-Electronic Conduction Properties of N-Type Polymers. *Adv. Funct. Mater.* **31**, 2008718 (2021).
11. Ohayon, D. *et al.* Influence of Side Chains on the n-Type Organic Electrochemical Transistor Performance. *ACS Appl. Mater. Interfaces* **13**, 4253–4266 (2021).
12. Samuel, J. J. *et al.* Single-Component CMOS-Like Logic using Diketopyrrolopyrrole-Based Ambipolar Organic Electrochemical Transistors. *Adv. Funct. Mater.* **31**, 2102903 (2021).
13. Feng, K. *et al.* Cyano-Functionalized n-Type Polymer with High Electron Mobility for High-Performance Organic Electrochemical Transistors. *Adv. Mater.* **34**, 2201340 (2022).
14. Li, P., Shi, J., Lei, Y., Huang, Z. & Lei, T. Switching p-type to high-performance n-type organic electrochemical transistors via doped state engineering. *Nat. Commun.* **13**, 5970 (2022).
15. Parr, Z. S. *et al.* From p- to n-Type Mixed Conduction in Isoindigo-Based Polymers through Molecular Design. *Adv. Mater.* **34**, 2107829 (2022).
16. Wang, Y. *et al.* Green Synthesis of Lactone-Based Conjugated Polymers for n-Type Organic Electrochemical Transistors. *Adv. Funct. Mater.* **32**, 2111439 (2022).
17. Wu, X. *et al.* All-Polymer Bulk-Heterojunction Organic Electrochemical Transistors with Balanced Ionic and Electronic Transport. *Adv. Mater.* **34**, 2206118 (2022).
18. Wu, H.-Y. *et al.* Influence of Molecular Weight on the Organic Electrochemical Transistor Performance of Ladder-Type Conjugated Polymers. *Adv. Mater.* **34**, 2106235 (2022).
19. Cong, S. *et al.* Donor Functionalization Tuning the N-Type Performance of Donor–Acceptor Copolymers for Aqueous-Based Electrochemical Devices. *Adv. Funct. Mater.* **32**, 2201821 (2022).
20. Wang, Y. *et al.* Acceptor Functionalization via Green Chemistry Enables High-Performance n-Type Organic Electrochemical Transistors for Biosensing, Memory Applications. *Adv. Funct. Mater.* **34**, 2304103 (2024).
21. Ohayon, D. *et al.* Salts as Additives: A Route to Improve Performance and Stability of n-Type Organic Electrochemical Transistors. *ACS Mater. Au* **3**, 242–254 (2023).

22. Chen, J. *et al.* Backbone coplanarity manipulation via hydrogen bonding to boost the n-type performance of polymeric mixed conductors operating in aqueous electrolyte. *Mater. Horiz.* **10**, 607–618 (2023).
23. Ma, S. *et al.* Sequential Cyanation of Polythiophenes: Tuning Charge Carrier Polarity in Organic Electrochemical Transistors. *Adv. Electron. Mater.* **9**, 2300207 (2023).
24. Duan, J. *et al.* Highly Efficient Mixed Conduction in a Fused Oligomer n-Type Organic Semiconductor Enabled by 3D Transport Pathways. *Adv. Mater.* **35**, 2300252 (2023).
25. Yu, Y. *et al.* n-Type Glycolated Imide-Fused Polycyclic Aromatic Hydrocarbons with High Capacity for Liquid/Solid-Electrolyte-based Electrochemical Devices. *Adv. Funct. Mater.* **33**, 2300012 (2023).
26. Duan, J. *et al.* Electron-Deficient Polycyclic Molecules via Ring Fusion for n-Type Organic Electrochemical Transistors. *Angew. Chem. Int. Ed.* **62**, e202213737 (2023).
27. Liu, K.-K. *et al.* J-Type Self-Assembled Supramolecular Polymers for High-Performance and Fast-Response n-Type Organic Electrochemical Transistors. *Adv. Funct. Mater.* **33**, 2300049 (2023).
28. Yang, W. *et al.* High-Performance n-Type Polymeric Mixed Ionic-Electronic Conductors: The Impacts of Halogen Functionalization. *Adv. Mater.* **36**, 2305416 (2024).
29. Wu, W. *et al.* Selenophene Substitution Enabled High-Performance n-Type Polymeric Mixed Ionic-Electronic Conductors for Organic Electrochemical Transistors and Glucose Sensors. *Adv. Mater.* **36**, 2310503 (2024).
30. Zeglio, E. *et al.* Mixing Insulating Commodity Polymers with Semiconducting n-type Polymers Enables High-Performance Electrochemical Transistors. *Adv. Mater.* **36**, 2302624 (2024).
31. Wu, X. *et al.* Stable n-Type Perylene Derivative Ladder Polymer with Antiambipolarity for Electrically Reconfigurable Organic Logic Gates. *Adv. Mater.* **36**, 2308823 (2024).
32. Zhang, C. *et al.* Polythiophenes for High-Performance N-type Organic Electrochemical Transistors. *Adv. Funct. Mater.* **35**, 2419706 (2025).
33. Inal, S. *et al.* A High Transconductance Accumulation Mode Electrochemical Transistor. *Adv. Mater.* **26**, 7450–7455 (2014).
34. Giovannitti, A. *et al.* Controlling the mode of operation of organic transistors through side-chain engineering. *Proc. Natl. Acad. Sci.* **113**, 12017–12022 (2016).
35. Nielsen, C. B. *et al.* Molecular Design of Semiconducting Polymers for High-Performance Organic Electrochemical Transistors. *J. Am. Chem. Soc.* **138**, 10252–10259 (2016).
36. Inal, S., Malliaras, G. G. & Rivnay, J. Benchmarking organic mixed conductors for transistors. *Nat. Commun.* **8**, 1767 (2017).
37. Li, P. & Lei, T. Molecular design strategies for high-performance organic electrochemical transistors. *J. Polym. Sci.* **60**, 377–392 (2022).
38. Kim, S.-M. *et al.* Influence of PEDOT:PSS crystallinity and composition on electrochemical transistor performance and long-term stability. *Nat. Commun.* **9**, 3858 (2018).
39. Savagian, L. R. *et al.* Balancing Charge Storage and Mobility in an Oligo(Ether) Functionalized Dioxythiophene Copolymer for Organic- and Aqueous- Based Electrochemical Devices and Transistors. *Adv. Mater.* **30**, 1804647 (2018).
40. Flagg, L. Q. *et al.* Polymer Crystallinity Controls Water Uptake in Glycol Side-Chain Polymer Organic Electrochemical Transistors. *J. Am. Chem. Soc.* **141**, 4345–4354 (2019).
41. Wang, Y. *et al.* Hybrid Alkyl–Ethylene Glycol Side Chains Enhance Substrate Adhesion and Operational Stability in Accumulation Mode Organic Electrochemical Transistors. *Chem. Mater.* **31**, 9797–9806 (2019).

42. Schmode, P. *et al.* High-Performance Organic Electrochemical Transistors Based on Conjugated Polyelectrolyte Copolymers. *Chem. Mater.* **31**, 5286–5295 (2019).
43. Moser, M. *et al.* Side Chain Redistribution as a Strategy to Boost Organic Electrochemical Transistor Performance and Stability. *Adv. Mater.* **32**, 2002748 (2020).
44. Giovannitti, A. *et al.* Energetic Control of Redox-Active Polymers toward Safe Organic Bioelectronic Materials. *Adv. Mater.* **32**, 1908047 (2020).
45. Savva, A. *et al.* Balancing Ionic and Electronic Conduction for High-Performance Organic Electrochemical Transistors. *Adv. Funct. Mater.* **30**, 1907657 (2020).
46. Jia, H. *et al.* Engineering donor–acceptor conjugated polymers for high-performance and fast-response organic electrochemical transistors. *J. Mater. Chem. C* **9**, 4927–4934 (2021).
47. Wang, Y. *et al.* The effect of the donor moiety of DPP based polymers on the performance of organic electrochemical transistors. *J. Mater. Chem. C* **9**, 13338–13346 (2021).
48. Luo, X. *et al.* Designing Donor–Acceptor Copolymers for Stable and High-Performance Organic Electrochemical Transistors. *ACS Macro Lett.* **10**, 1061–1067 (2021).
49. Hallani, R. K. *et al.* Regiochemistry-Driven Organic Electrochemical Transistor Performance Enhancement in Ethylene Glycol-Functionalized Polythiophenes. *J. Am. Chem. Soc.* **143**, 11007–11018 (2021).
50. Moser, M. *et al.* Polaron Delocalization in Donor–Acceptor Polymers and its Impact on Organic Electrochemical Transistor Performance. *Angew. Chem. Int. Ed.* **60**, 7777–7785 (2021).
51. Wu, X. *et al.* Enhancing the Electrochemical Doping Efficiency in Diketopyrrolopyrrole-Based Polymer for Organic Electrochemical Transistors. *Adv. Electron. Mater.* **7**, 2000701 (2021).
52. Krauss, G. *et al.* Polydiketopyrrolopyrroles Carrying Ethylene Glycol Substituents as Efficient Mixed Ion-Electron Conductors for Biocompatible Organic Electrochemical Transistors. *Adv. Funct. Mater.* **31**, 2010048 (2021).
53. Wu, H.-Y. *et al.* Stable organic electrochemical neurons based on p-type and n-type ladder polymers. *Mater. Horiz.* **10**, 4213–4223 (2023).
54. E. Chen, S. *et al.* Impact of varying side chain structure on organic electrochemical transistor performance: a series of oligoethylene glycol-substituted polythiophenes. *J. Mater. Chem. A* **10**, 10738–10749 (2022).
55. Hidalgo Castillo, T. C. *et al.* Simultaneous Performance and Stability Improvement of a p-Type Organic Electrochemical Transistor through Additives. *Chem. Mater.* **34**, 6723–6733 (2022).
56. Lan, L. *et al.* Facilely Accessible Porous Conjugated Polymers toward High-Performance and Flexible Organic Electrochemical Transistors. *Chem. Mater.* **34**, 1666–1676 (2022).
57. Cong, S. *et al.* Tunable control of the performance of aqueous-based electrochemical devices by post-polymerization functionalization. *Mater. Horiz.* **10**, 3090–3100 (2023).
58. Bardagot, O. *et al.* Over Tenfold Increase in Current Amplification Due to Anisotropic Polymer Chain Alignment in Organic Electrochemical Transistors. *Adv. Mater.* 2420323 (2025).
59. Liao, H. *et al.* High Performance Organic Mixed Ionic-Electronic Polymeric Conductor with Stability to Autoclave Sterilization. *Angew. Chem.* **137**, e202416288 (2025).
60. Chen, W.-M. *et al.* A fully integrated 8-channel closed-loop neural-prosthetic CMOS SoC for real-time epileptic seizure control. *IEEE J. Solid-State Circuits* **49**, 232-247, (2013).
61. O'Leary, G. *et al.* in *Proc. IEEE Int. Solid-State Circuits Conf. (ISSCC), 2020.* 402-404 (IEEE).
62. Cheng, C.-H. *et al.* A fully integrated 16-channel closed-loop neural-prosthetic CMOS SoC with wireless power and bidirectional data telemetry for real-time efficient human epileptic seizure control. *IEEE J. Solid-State Circuits* **53**, 3314-3326, (2018).

63. O'Leary, G., Groppe, D. M., Valiante, T. A., Verma, N. & Genov, R. NURIP: Neural interface processor for brain-state classification and programmable-waveform neurostimulation. *IEEE J. Solid-State Circuits* **53**, 3150-3162, (2018).
64. Wang, Y. *et al.* A closed-loop neuromodulation chipset with 2-level classification achieving 1.5-Vpp CM interference tolerance, 35-dB stimulation artifact rejection in 0.5 ms and 97.8%-sensitivity seizure detection. *IEEE Trans. Biomed. Circuits Syst.* **15**, 802-819, (2021).
65. Liu, Z. *et al.* Neural signal analysis with memristor arrays towards high-efficiency brain–machine interfaces. *Nat. Commun.* **11**, 4234, (2020).
66. Zhao, Z., Cea, C., Gelinas, J. N. & Khodagholy, D. Responsive manipulation of neural circuit pathology by fully implantable, front-end multiplexed embedded neuroelectronics. *Proc. Natl. Acad. Sci. U.S.A.* **118**, e2022659118, (2021).
